# The effect of age on the heart rate variability of healthy subjects

**DOI:** 10.1371/journal.pone.0255894

**Published:** 2021-10-08

**Authors:** Leopoldo Garavaglia, Damián Gulich, Magdalena M. Defeo, Julieta Thomas Mailland, Isabel M. Irurzun

**Affiliations:** 1 Centro Científico Tecnológico (CCT) La Plata- CONICET, Instituto de Investigaciones Fisicoquímicas Teóricas y Aplicadas (INIFTA), Facultad de Ciencias Exactas, Universidad Nacional de La Plata, La Plata, República Argentina; 2 Centro de Investigaciones Opticas, La Plata, República Argentina; 3 Hospital Interzonal General de Agudos “Prof. Dr. Rodolfo Rossi”, La Plata, República Argentina; University of Milan, ITALY

## Abstract

In this work we study the characteristics of heart rate variability (HRV) as a function of age and gender. Our analysis covers a wider age range than that studied so far. It includes results previously reported in the literature and reveals behaviours not reported before. We can establish basic scale relationships in different HRV measurements. The mean value of the RR intervals shows a power-law behaviour independent of gender. Magnitudes such as the standard deviation or pNN50 show abrupt changes at around the age of 12 years, and above that age they show gender dependence, which mainly affects short-time (or high frequency) scales. We present a unified analysis for the calculation of the non-linear *α* and *β* parameters. Both parameters depend on age; they increase in the extremes of life and reach a minimum at around one year of age. These gender-independent changes occur at low frequencies and in scale ranges that depend on age. The results obtained in this work are discussed in terms of the effects of basal metabolic rate, hormonal regulation, and neuronal activity on heart rate variability. This work finally discusses how these findings influence the interpretation of HRV measurements from records of different lengths.

## Introduction

Heart rate variability (HRV) is the physiological variation in the duration of cardiac cycles [1, 2]. Research into HRV began with the emergence of modern signal processing in the 1960s and 1970s, and has rapidly expanded in more recent times [3]. HRV is mainly controlled by the autonomic nervous system (ANS) through the interplay of sympathetic and parasympathetic neural activity especially at the sinus node [4, 5]. The autonomic nervous system interacts with receptors on the sinoatrial node (SAN) and the relative contributions of the ANS and SAN to HRV have been elucidated in [6, 7]. In general, HRV is influenced by many several factors such as chemical, hormonal and neural modulations, circadian changes, exercise, emotions, posture, and preload. The heart rate adaptation to changing factors is carried out by the activity of a variety of regulatory subsystems, and results in a complex linear and non-linear time behaviour, which changes with age and pathological conditions.

Several studies demonstrated age-related and gender-related variations in long-term HRV characteristics. It was reported that autonomic activities diminish with age in both genders and that gender-related variation in parasympathetic regulation decreases after the age of 50 years [8–14]. The hormonal influence on autonomic activity was analysed in [15–20]. Some investigators found that cardiac vagal activity declined during the luteal phase, and others reported that no changes in cardiovascular autonomic control occurred during the menstrual cycle of healthy fertile women. These studies were conducted on small populations and using HRV series too short to compare the results with ours.

HRV characteristics were proposed as predictors of the risk of premature mortality after myocardial infarction or development of congestive heart failure, also as diagnosis tools of autonomic dysfunction in diabetes, and as non-invasive tools for estimating the autonomic modulation of the cardiovascular system during stress, relaxation, or assessment of the effects of physical training on fitness level. For these reasons, the interest in HRV is growing both in clinical and physiological studies [21–28]. The importance of HRV as an index of the functional status of physiological control systems is currently recognized.

To compute HRV characteristics, many mathematical methods have been developed, which may be grouped into statistical, spectral, graphical, non-linear, complexity or information based [29–32].

In summary, the scientific and medical community has made a huge effort to obtain reliable measurements of the HRV characteristics in normal and pathological conditions. The study of the dependence of HRV on age and gender in a healthy population reveals the influence of factors such as body mass, basal metabolic rate, hormonal regulation, neuronal activity, etc. It also allows characterizing a control group to reference deviations associated with various pathologies.

There are many studies in the literature that together explore an age range of 1-99 years [2, 33–42].

In this paper we present a study that covers an age range of 0.08 (1 month)-99 years, increasing the age range by slightly more than a power of ten. The difference is significant for revealing scaling behaviours in the HRV measurements. In our analysis we include data obtained from [2, 33–35] that are representative of the age range 1-99 years and sufficient for the purpose of this work.

Finley et al. presented calculations of low frequency (LF), high frequency (HF), and total power indices for the range of 0.08-24 years [43]. LF and HF indices are not analysed in our work, and total power is equivalent to the standard deviation of HRV, whose dependence on age is discussed in this work. In that study, however, the data are grouped into age ranges, that is, age is not considered a continuous variable. Van den Berg et al. [44] presented a very large study including some of the indices that we calculate, but they examined 10-second electrocardiograms, which are too short to calculate scale factors by power and multifractal analyses, as we discuss in the last section.

We calculate indices in the time domain and scale factors (*α* and *β*) derived from both the multifractal and the power analyses, and a unified analysis of them is presented. There is a mathematical relationship between *α* and *β*, and our unified analysis suggests that the scale ranges depend on age. We also discuss *α* and *β* dependences on age in terms of the influence of neuronal activity on heart rate variability (due to either maturation of the autonomic nervous system or alterations in its interaction with the cells of the sinoatrial node).

Our work is limited to the analysis of the above-mentioned HRV characteristics [45, 46], but the methodological aspects reported in this work are general and should be considered to analyse any other measurements. By considering the HRV as a signal composed of multiple frequencies influenced by control mechanisms operating in different frequency ranges, there are two immediate aspects to consider: (i) the length of the HRV series is a variable coupled to the physiological variables, (ii) the HRV measurements must be determined in the same scale ranges in both the time and frequency domains. In other words, the length of the HRV series limits the scale range that can be analysed [47, 48]. There is a growing interest in using short and ultrashort HRV records because of their obvious clinical utility. To do this, it is necessary to understand the differences with 24-hour measurements, which have not yet been fully standardized. It is evident that by shortening the series, the frequencies under study are limited. A better understanding of the frequency composition of HRV and its dependence on age and gender can facilitate the use of short records.

This work is organised as follows: in Procedure we explain the methodology used, which is the same as that in [47, 48] and comparable to the one in [2, 33, 34]. The next section deals with the results; results of indices in the time domain are presented first, and then are results of scale factors *α* and *β*. The last section summarizes our conclusions.

## Procedure

We analysed Holter recordings from healthy subjects who were recruited as volunteers after an exhaustive interview and clinical examination. Approval for this study was granted by the Ethics Committee of the National University of La Plata (UNLP) for data protection and privacy. In accordance with the Declaration of Helsinki, prior to participation in the study, all subjects were informed about the study and gave their written consent (in the case of children under 16, their parent’s or legal guardian’s consent was required).

The study included participants without clinical symptoms of disease, who were not on medication and whose electrocardiograms (ECG) were normal according to the criteria summarized in Table 1 [47, 48].

**Table 1:** Criteria of normality for all Holter records obtained in the present work.

I	Minimum nighttime frequency>60/min
II	Nighttime pauses< 3s.
III	Ventricular extrasystoles < 100/24 h, without couplets, bursts, or polymorphism.
IV	Supraventricular extrasystoles < 100/24 h, without couplets, or bursts.
V	Absence of blocks or conduction disturbances.

Following these criteria, a total of 154 individuals were selected, who were aged between 1 month and 55 years (the age distribution is shown in Table 2).

**Table 2:** Age distribution of the 154 individuals included in the research.

Age range (x in years)	Number of patients
0 < *x* ≤ 0.17	16
0.17 < *x* ≤ 0.42	28
0.42 < *x* ≤ 0.67	17
0.67 < *x* ≤ 1.0	12
1.0 < *x* ≤ 2.0	14
2.0 < *x* ≤ 4.0	14
4.0 < *x* ≤ 7.0	13
7 < *x* ≤ 12	17
12 < *x* ≤ 30	15
30 < *x* ≤ 55	8

Holter monitoring was recorded for 24 h with digital three-channel DMS300 7 and DMS300 3A recorders, and Galix recorders, using 3M electrodes [49]. The Galix recorders had a programmable read in sampling rate of 512 and 1024 Hz, and a write out sampling rate of 128 Hz. The DMS recorders had a sampling rate of 1024 Hz per channel for signal-averaged electrocardiography (SAEG) analysis, a read in sampling rate of 512 Hz, and a write out sampling rate of 128 Hz in the other cases. The signals were analysed with Galix software, and CardioScan 10.0, 11.0 software for DMS recorders. The error in the RR interval determination was of about 8 ms (twice the error in the determination of the R peak).

The records of cardiac events were automatically detected and classified by the Holter software, and then examined and corrected by two cardiologists. We employed the quality criteria established in [47, 48] for all time series used in the present work. Also stationarity was evaluated, and surrogate analysis was performed as in [50–53].

Time series of a total of 195 healthy participants were analysed. Of these time series, 154 corresponded to the participants mentioned above, 13 to [54, 55], and 28 to [54, 56]. Participants from [54, 56] were aged from 20 to 74 years (the age distribution is shown in Table 3), and 50% of them were females. Holter monitoring was recorded for 24 h with digital three-channel DMS recorders, the signals were analysed with CardioScan (so the error in the RR interval determination was also 8 ms), and then examined and corrected by two cardiologists. The HRV time series were selected after the evaluation according to quality criteria established in [47, 48]. For comparison we also analysed data from [2, 33–35]. The characteristics of the population studied in [2, 34] are shown in Tables 4 and 5 respectively. In [33] two hundred sixty healthy subjects 10 to 99 years old (112 males, 148 females) were recruited (the age distribution is shown in Table 6). Healthy subjects were defined as those without clinical evidence of organic disease in terms of medical history, physical examination, rest 12-lead electrocardiogram, routine blood chemistry profiles, and complete blood count. Oral contraceptives and non-steroidal anti-inflammatory agents were the only medications allowed. Subjects who exhibited abnormalities in one or more of the aforementioned categories were excluded from the study. Holter monitoring was recorded for 24h and analysed with different three recorders and software, and randomly selected Holter tapes were cross-analysed with the use of all programs. HRV and HR determinations from identical tapes were within 10% of each other. Recordings <20 h in duration, and those in which nonsinus beats comprised 10% of the total number of beats, were excluded.

**Table 3:** Age distribution of the 41 individuals from [54–56].

Age range (x in years)	Number of patients
20 < *x* ≤ 40	14
40 < *x* ≤ 60	5
60 < *x* ≤ 74	22

**Table 4:** Clinical characteristics of the population used in [2].

	Females (n = 51)	Males (n = 49)
Age (years)	13.9 ± 3.7	12.3 ± 3.9
PQ (ms)	129.0 ± 14.7	129.9 ± 17.8
QRS (ms)	83.8 ± 8.6	87.2 ± 9.0
QTc (ms)	398.1 ± 16.7	387.6 ± 21.7
Supraventricular beats in 24h ECG (%)	(2.35 ± 9.3) × 10^−4^	(1.73 ± 6.5) × 10^−3^
Ventricular beats in 24h ECG (%)	(1.76 ± 5.19) × 10^−4^	0.205±1.425
Duration of ECG recording (min)	1287 ± 119	1313 ± 82

**Table 5:** Clinical, lifestyle and laboratory characteristics of the population used in [34].

	Males (n = 192)	Females (n = 202)
Age (years)	50 ± 6	51 ± 6
Body Mass Index (kg/m²)	26.4 ± 3.5	25.6 ± 4.0
Waist/hip ratio	0.91 ± 0.06	0.78 ± 0.05
Systolic blood pressure (mmHg)	147 ± 19	138 ± 20
Diastolic blood pressure (mmHg)	89 ± 11	82 ± 12
No smoking	123 (64%)	150 (74%)
Moderate smoking (<20/d)	57 (30%)	49 (24%)
Heavy smoking (>20/d)	12 (6%)	3 (2%)
Duration of smoking	0-46 years	0-57 years
No alcohol drinking	24 (13%)	96 (50%)
1-100g/week alcohol drinking	72 (37%)	37 (18%)
> 100g/week alcohol drinking	162 (80%)	3 (2%)
No physical activity	6 (3%)	57 (30%)
Mild physical activity	68 (35%)	61 (32%)
Moderate physical activity	7 (3%)	44 (22%)
Heavy physical activity	69 (34%)	82 (41%)
Personality type: Framingham	26 ± 5	22 ± 3
Personality type: Bortener	7 ± 3	28 ± 5
Personality type: Hostility	23 ± 3	7 ± 3
Fasting blood glucose (mmol/l)	4.4 ± 0.5	4.3 ± 0.4
2-hour blood glucose (mmol/l)	5.1 ± 1.5	5.1 ± 1.3
Fasting serum insulin (mU/l)	13 ± 9	9 ± 6
2-hour serum insulin (mU/l)	55 ± 53	54 ± 45
Total serum cholesterol (mmol/l)	5.78 ± 1.11	5.49 ± 1.00
HDL- cholesterol (mmol/l)	1.24 ± 0.30	1.57 ± 0.38
LDL- cholesterol (mmol/l)	3.75 ± 0.95	3.30 ± 0.90
VLDL- cholesterol (mmol/l)	0.43 ± 0.29	0.28 ± 0.20
Serum triglycerides (mmol/l)	1.51 ± 0.76	1.13 ± 0.60
Left ventricular mass #	221 ± 53	154 ± 35
Left ventricular mass index #	112 ± 26	91 ± 19
Fractional shortening (%) #	34 ± 6	35 ± 5

^#^ n = 193 women, n = 166 men.

**Table 6:** Age and gender distribution of the individuals from [33].

Age range (x in years)	Male	Female
10 < *x* ≤ 19	16	14
20 < *x* ≤ 29	16	26
30 < *x* ≤ 39	19	20
40 < *x* ≤ 49	25	40
50 < *x* ≤ 59	11	11
60 < *x* ≤ 69	10	10
70 < *x* ≤ 79	9	12
80 < *x* ≤ 99	6	15

In total, we evaluated data from about 700 healthy subjects aged between 1 month and 99 years.

The age distributions in Tables 2, 3 and 6 are given as histograms dividing the population into subgroups. However, in our analysis we consider the age as a continuous variable and therefore we did not carry out an analysis of the size effect in these groups. The effect of size is considered in linear regressions as explained below.

The following linear indices in the time domain were calculated: the mean value of the RR intervals (<*RR*>), the standard deviation of the RR intervals (*SD*_*RR*_), the square root of the mean squared sum of differences of successive RR intervals (*rMSSD*_*RR*_), and the percentage of the intervals that vary by more than 50 ms from the previous interval (*pNN*50). These indices were calculated using the complete series, without detrending procedure. As we will discuss below, the length of the series affects its frequency composition (also does the detrending procedure) and this fact must be considered when comparing results obtained from short and long series. Our series are long enough so that the mentioned indices have values independent of the length of the series [47, 48].

As we mentioned in the Introduction, there are other widely used HRV indices, for example total power, and the LF and HF indices [33, 34, 43, 44]. The total power is equivalent to *SD*_*RR*_. The LF and HF indices are calculated over specific frequency ranges. One of our conclusions points to the need to improve the definition of the frequency ranges studied, which would be dependent on age. For this reason we focus on the scale factors and the relationship between them, as explained below.

It is known that HRV follows a power-law behaviour in the frequency domain (1/beat), which is manifested in the power spectrum dependence on frequency as

$$\begin{array}{c} S\left(f\right)\alpha f^{-\beta } \end{array}$$ (1)

The values for *β* were determined in this work by averaging the power spectra of successive time series segments, each consisting of 4096 datapoints. This procedure, which allows eliminating high frequency fluctuations, is detailed in [47]. Power spectra were calculated using fast Fourier transform (FFT) after normalizing the series to zero mean value. The method to calculate *β* used the optimal range for each participant (roughly in the range 2 × 10^−4^–5 × 10^−2^Hz) [47]. No detrending procedure was used. In the next section we compare our results with those obtained in [34]. In [34]the slope (*β*) was calculated from the frequency range of 10^−4^ to 10^−2^ Hz from the 24-hour ECG recordings. The point power spectrum was logarithmically smoothed in the frequency domain, and the power was integrated into bins spaced 0.0167 log(Hz) apart. A line-fitting algorithm of log(power) on log(frequency) was then applied to the power spectrum between 10^−4^ and 10^−2^ Hz, and the slope of this line was calculated.

The detrended fluctuation analysis (DFA) is widely used to characterize the fractal dynamics of a system from which a time series has been measured [57, 58], and it is the most popular approach to detect the presence of long-term memory in data [59]. By the DFA technique a scale exponent *α* is determined, which is related to *β* as: *β* = 2*α*−1. However, we rarely find a verification of this relationship in scientific papers. There are several reasons for this omission. The value of *α* depends on the scale ranges chosen to calculate it, and there is no agreement on such ranges. The scale ranges for calculating *β* should be compatible with those used for *α*, but the reliable calculation of the power spectrum usually requires more data.

The DFA follows five steps [60–62]

- Step 1: given a time series *S* = {*x*_*t*_, *t* = 1, …, *N*}, with *N* being the number of equidistant observations, the cumulative sum of the data $Y(k)=\sum_{t=1}^{k}(x_{t}-\langle x\rangle )$, with *k* = 1, …, *N* and $\langle x\rangle =(\sum_{t=1}^{N}x_{t})/N$, is considered.
- Step 2: this profile is divided into ⎣*N*/*s*⎦ non-overlapping windows of equal length *s* (⎣*a*⎦ denotes the largest integer less than or equal to *a*).
- Step 3: a local polynomial fit *y*_*ν*,*m*_(*k*) of degree *m* is fitted to the profile for each window *ν* = 1, …, ⎣*N*/*s*⎦. The degree of the polynomial can be varied to eliminate constant (*m* = 0), linear (*m* = 1), quadratic (*m* = 2) or higher order trends of the profile. It is customary to indicate the degree of detrending by including it in the title of the technique (DFA- *m*).
- Step 4: the variance of the detrended time series is evaluated by averaging over all datapoints *k* in each segment *ν*, $F_{m}^{2}(\nu ,s)=(1/s)\sum_{k=1}^{s}{\left\{Y[(\nu -1)s+k]-y_{\nu ,\;m}(k)\right\}}^{2}$, for *ν* = 1, …, ⎣*N*/*s*⎦.
- Step 5: the DFA fluctuation function is obtained by averaging over all segments and taking the square root, $F_{m}(s)={\left\{(1/\lfloor N/s\rfloor )\sum_{\nu =1}^{\left\lfloor N/s\right\rfloor }[F_{m}^{2}(\nu ,s)]\right\}}^{1/2}$.

By repeating this procedure for different values of *s*, the *s*-dependence of *F*_*m*_ s is obtained.

If the time series has long-range power-law correlations, *F*_*m*_(*s*) scales as

$$\begin{array}{c} F_{m}(s)∼s^{\alpha } \end{array}$$ (2)

for a certain range of *s* [62]. The scaling exponent *α* is estimated by the slope of the best linear regression in a double logarithmic plot. The long-range correlations embedded in the time series are quantified by this exponent: if *α* > 1/2, consecutive increments tend to have the same sign, so the processes are *persistent*. If *α* < 1/2, consecutive increments are more likely to have opposite signs, and it is said that the processes are *anti-persistent*. For uncorrelated data *α* = 1/2 is obtained [63].

We calculated the fluctuation functions *F*_2_(*s*) with detrending degrees *m* = 1 − 4 (scales *s* ranging from 5 to 200 datapoints). For *m* = 3, 4, detrending artifacts appear, and therefore *m* values should be limited to 1 or 2 for a correct estimation of *α*. To compare with previous results, we used three sets of scales *s* to fit all fluctuation functions, thus finding three scaling exponents (Eq (2)):

- *α*_0_ for scales 5 ≤ *s* ≤ 10 (very high frequency exponent) [34]
- *α*_1_ for scales 10 ≤ *s* ≤ 50 (high frequency exponent) [35]
- *α*_2_ for scales 50 ≤ *s* ≤ 200 (medium frequency exponent) [35]

Note that the scale ranges used to calculate *α*_*i*_(*i* = 0, 1, 2) are somewhat different from those used in Peng’s original article [58]. However, these differences do not imply significant changes in the values of *α*_*i*_ or modify the conclusions of this work. We used the scales that were employed in the papers whose data were used for comparison. In fact, in this work we show that the scale ranges are dependent on age and must be determined consistently in both the time and frequency domains.

In Results the relationships of the linear indices with age are presented. Age is considered as a continuous variable. A logarithmic transformation is then applied to both the independent and dependent variables to reveal scale relationships between the variables, which is manifested as a straight line in a log-log plot. The scale factors are determined by performing a linear regression fit using the least squares method, and the significance of the results is evaluated by an analysis of variance (ANOVA). The size effects can be evaluated either through Cohen’s d index or the regression coefficient (R). We used R in this work, and the values obtained indicate that the size effects are negligible in the relationships proposed in this work (Eqs 3–6). Other adjustments previously proposed in the scientific literature were also evaluated, and the ANOVA tables showed that the scale relationships were the most acceptable. The revelation of the scale relationships in this work is a consequence of the widening of the age range and the consideration of age as a continuous variable. When analysing the influence of gender, we performed a covariance analysis (ANCOVA). The size effects were also negligible in these cases.

The analyses of the linear indices included data from other studies that show differences in the methodology or in the population studied. As far as we could verify, these differences did not significantly affect our results (see Results).

**Fig 1:**
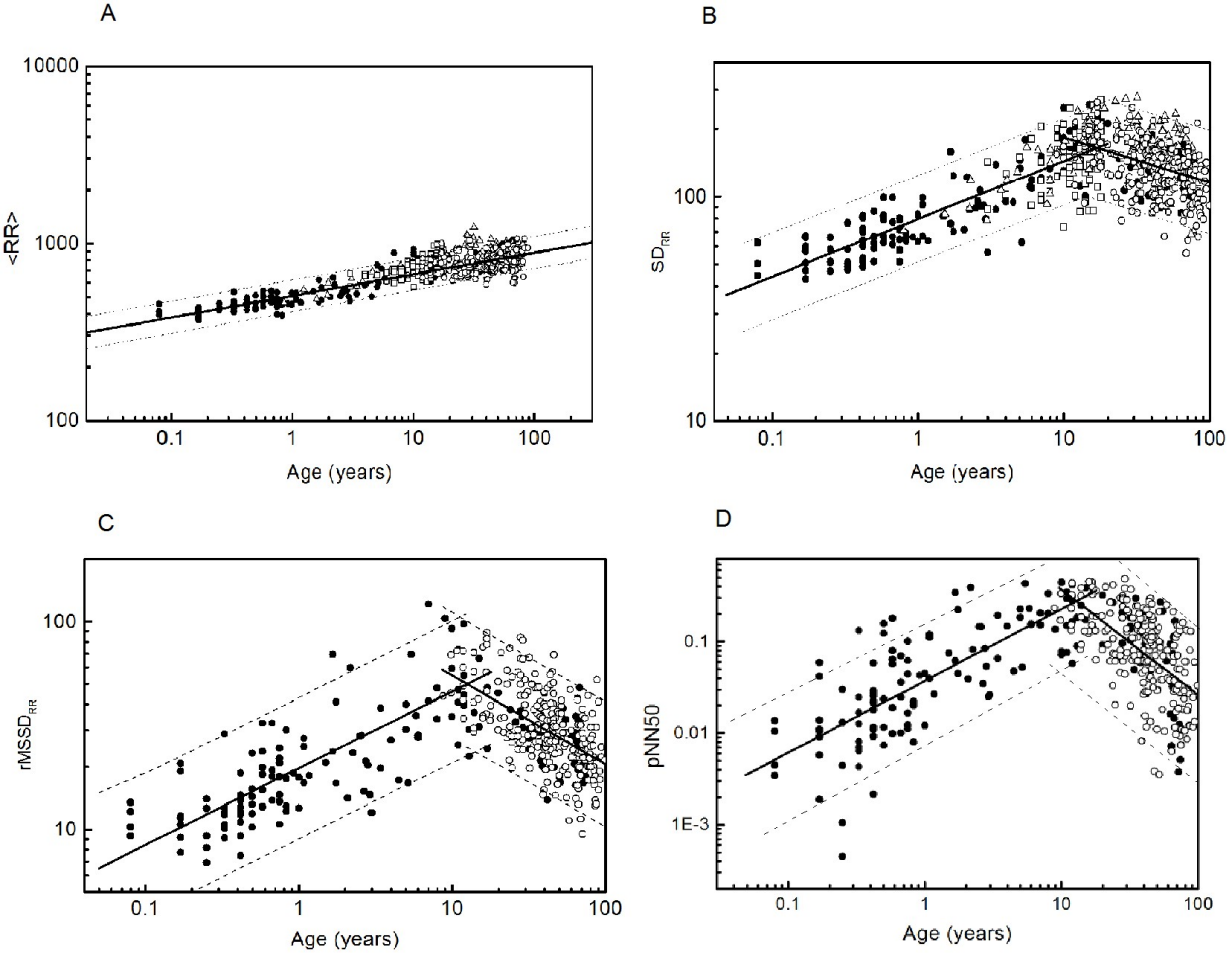
Age dependence of some time-domain indices: (A)<*RR*>, (B) *SD*_*RR*_, (C) *rMSSD*_*RR*_, (D) *pNN*50 Filled circles correspond to data obtained in this work, open squares to data from [2], open circles to data from [33], and open triangles to data from [34]. Dashed lines indicate the 95% prediction intervals.

Finally, although age is considered a continuous variable, our study is cross-sectional. In the proposed adjustments for the linear indices we then included the prediction intervals (see Fig 1). A prediction interval is an estimate of a range of values in which a future observation will occur with a certain probability, given what has already been observed. Thus, in a hypothetical longitudinal study, the indices of a healthy individual should evolve within the prediction intervals.

**Fig 2:**
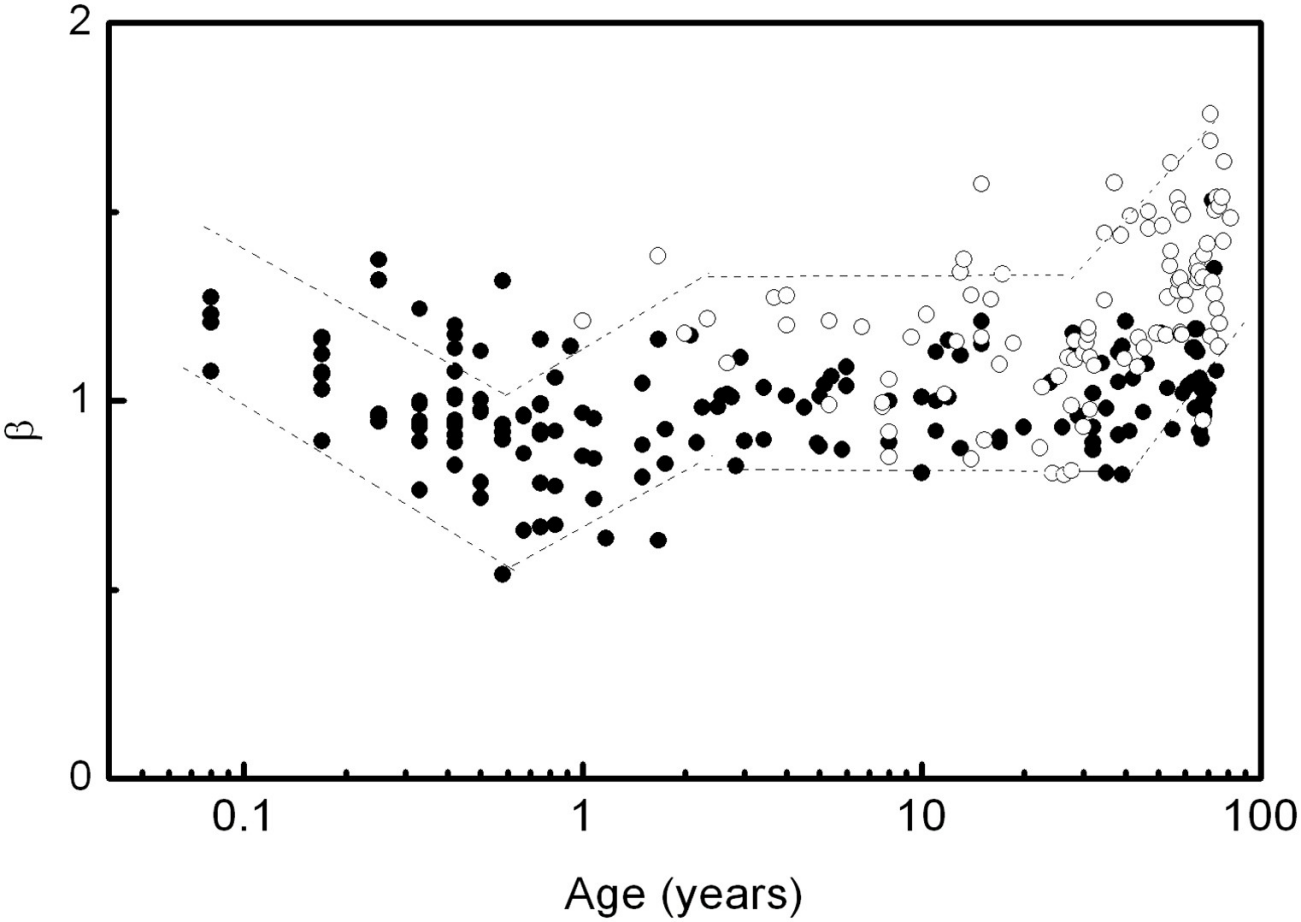
*β* dependence on age. Filled circles correspond to data obtained in this work, open circles to data from [34]. Dashed lines indicate the approximate 95% prediction interval (see Procedure).

**Fig 3:**
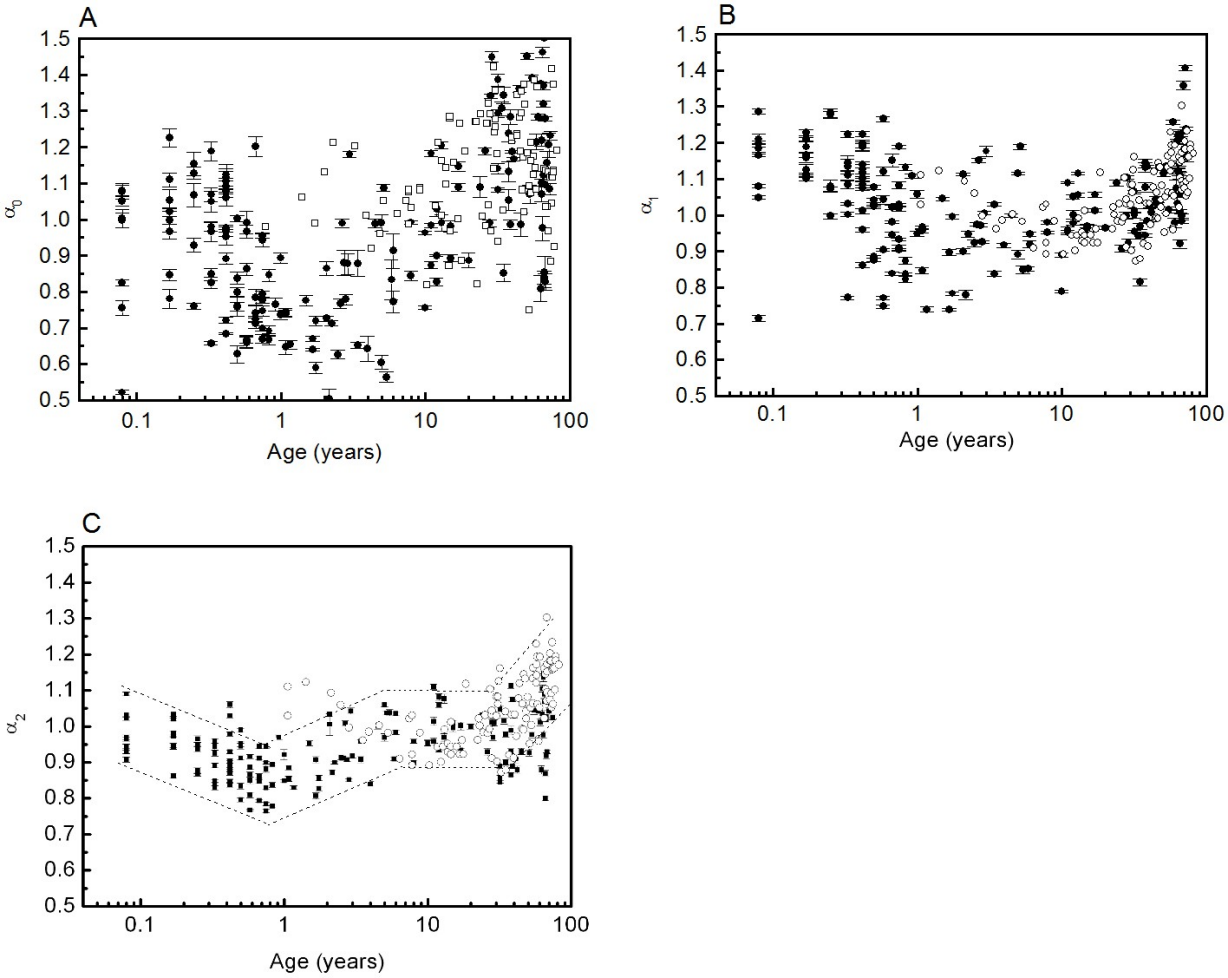
*α*_0_ (A), *α*_1_(B), and *α*_2_(C) dependence on age. Filled circles correspond to data obtained in this work, and open squares and open circles correspond to data from [34], for *α*_0_ and *α*_1_ respectively. Note that in [34]*α*_1_ was determined for *s* > 11. In (C) the dashed lines indicate the approximate 95% prediction interval (see Procedure).

The dependence of the *α* and *β* scale factors on age is presented in linear-log plots. In these cases, growth trends and the existence of critical points (maximum or minimum) are analysed, and the application of a logarithmic transformation to age was done in order to better visualize the behaviour at small age values. We do not propose a specific functional form for *α* and *β* dependence on age in this work. The prediction intervals could be roughly calculated by locally approximating the function by line segments. We did this for *β* in Fig 2 and *α*_2_ in Fig 3 for the reasons we discuss in Results.

## Results

### Time-domain indices

Fig 1 shows the dependence of time domain indices on age, including data from [2, 33, 34].

Data show good agreement among these indices, validating the general treatment of the measurements. Our data cover a wider age range, revealing novel tendencies. While <*RR*> exhibits a monotonic behaviour, *SD*_*RR*_, *rMSSD*_*RR*_, and *pNN*50 show an abrupt change at about the age of 12 years. In Fig 1 log-log scales reveal the existence of scale laws, which appear as linear behaviours after a logarithmic transformation. Results have been fitted to scaling laws as follows:

$$\begin{array}{c} <RR>=\left(505\pm 5\right)x^{\left(0.122\pm 0.004\right)} \end{array}$$ (3)

$$\begin{array}{c} SD_{RR}=\{\begin{array}{c} \left(80\pm 1\right)x^{\left(0.26\pm 0.01\right)}\begin{array}{ccccc} & & & & x<12 \end{array}\\ \left(290\pm 20\right)x^{\left(-0.20\pm 0.02\right)}\begin{array}{ccccc} & & & & x>12 \end{array} \end{array}\} \end{array}$$ (4)

$$\begin{array}{c} rMSSD_{RR}=\{\begin{array}{c} \left(18,6\pm 0.2\right)x^{\left(0.34\pm 0.02\right)}\begin{array}{ccccc} & & & & x<12 \end{array}\\ \left(166\pm 11\right)x^{\left(-0.46\pm 0.04\right)}\begin{array}{ccccc} & & & & x>12 \end{array} \end{array}\} \end{array}$$ (5)

$$\begin{array}{c} pNN50=\{\begin{array}{c} \left(0.037\pm 0.001\right)x^{\left(0.78\pm 0.07\right)}\begin{array}{ccccc} & & & & x<12 \end{array}\\ \left(5\pm 1\right)x^{\left(-1.1\pm 0.1\right)}\begin{array}{ccccc} & & & & x>12 \end{array} \end{array}\} \end{array}$$ (6)

where *x* is the age in years, and the statistics of the adjustments are shown in Table 7.

**Table 7:** Statistics of Eqs 3, 4, 5 and 6.

Index	Statistics			Index	Statistics		
<*RR*>	Eq 3			*rMSSD_RR_*	Eq 5	x<12	x>12
	N	560			N	116	268
	R	0.92			R	0.81	−0.54
	sd	0.045			sd	0.17	0.16
	p<	10^−4^			p<	10^−4^	10^−4^
*SD*_*RR*_	Eq 4	x<12	x>12	*pNN*50	Eq 6	x<12	x>12
	N	177	435		N	129	257
	R	0.86	−0.40		R	0.82	−0.56
	sd	0.1	0.1		sd	0.37	0.41
	p<	10^−4^	10^−4^		p<	10^−4^	10^−4^

The calculation includes data extracted from [2, 33, 34] as in Fig 1. N is the number of data, sd is the standard deviation, R is the correlation coefficient, and p is the Student’s t-parameter.

The threshold *x* = 12 was determined for *SD*_*RR*_, and the same value was assumed valid for *rMSSD*_*RR*_and *pNN*50. We made linear fits by changing the cut-off value, and *x* = 12 was chosen because the linear fits on both sides were the best. Though the power-law adjustments for ages above 12 years are statistically worse than those for ages below 12 years, they are still better than or equal to other linear or quadratic fits performed on the same data sets.

Quadratic fits over the complete data set have been tested, but they were statistically less reliable than those presented in this work. Table 8 shows the analysis of variance (ANOVA) tables of different fits performed in this work.

**Table 8:** ANOVA tables for different fits over the complete data set tested in this work.

Index	ANOVA Table						
*SD*_*RR*_	Linear-Linear Scale		Log-Log Scale				
	Quadratic polynomial		Eq 4			Quadratic polynomial	
				x<12	x>12		
	SSE	1.08 × 10^6^	SSE	1.8	5.8	SSE	8.5
	SST	1.4 × 10^6^	SST	6.5	6.9	SST	16.8
	MSM	146515	MSM	4.7	1.1	MSM	4.15
	MSE	1773	MSE	0.01	0.01	MSE	0.014
*rMSSD*_*RR*_	Linear-Linear Scale		Log-Log Scale				
	Quadratic polynomial		Eq 5			Quadratic polynomial	
				x<12	x>12		
	SSE	107360	SSE	3.3	6.5	SSE	11.7
	SST	121000	SST	9.5	9.1	SST	20.7
	MSM	6820	MSM	6.2	2.6	MSM	4.5
	MSE	281	MSE	0.029	0.024	MSE	0.03
*pNN*50	Linear-Linear Scale		Log-Log Scale				
	Quadratic polynomial		Eq 6			Quadratic polynomial	
				x<12	x>12		
	SSE	40	SSE	15.4	44.6	SSE	63.4
	SST	46	SST	46.1	65.2	SST	114.2
	MSM	56	MSM	30.7	20.6	MSM	25.35
	MSE	2.1	MSE	0.13	0.17	MSE	0.17

SST and SSE are the sum of squares total and error, MSM, and MSE are the mean squares model and error.

We find slight differences by gender only for ages over 12. Table 9 summarizes the power-law parameters for each case. We performed an analysis of covariance (ANCOVA) to assess the influence of gender and the p-values of the covariate are shown in the last column.

**Table 9:** Power law adjustments by gender for participants over 12 years old.

Index	Male		Female		Covariate p-value
*SD*_*RR*_	Eq.	398(8)*x*^−0.28(4)^	Eq.	229(4)*x*^−0.17(4)^	*p* < 10^−3^
	N	123	N	148	
	R	−0.55	R	−0.36	
	sd	0.1	sd	0.1	
	p<	10^−4^	p<	10^−4^	
*rMSSD*_*RR*_	Eq.	200(20)*x*^−0.54(7)^	Eq.	160(20)*x*^−0.45(6)^	*p* < 10^−3^
	N	125	N	143	
	R	−0.57	R	−0.51	
	sd	0.14	sd	0.16	
	p<	10^−4^	p<	10^−4^	
*pNN*50	Eq.	12(2)*x*^−1.4(2)^	Eq.	1.7(1)*x*^−0.8(1)^	*p* < 10^−4^
	N	124	N	133	
	R	−0.63	R	−0.43	
	sd	0.36	sd	0.46	
	p<	10^−4^	p<	10^−4^	

The calculation includes data extracted from [33, 35]. The numbers between parentheses indicate the error of the estimates, N is the number of data, R is the correlation coefficient, sd the standard deviation, and p is the Student’s t-parameter.

Note that the R values do not improve when discriminating the population by gender. We also made adjustments for x > 12 only for the population in [33, 35], and the values of R were similar. A similar result was reported in [34]. It is evident that there is great individual variability in HRV indices in the population over 12, which we discuss in the last section.

### Scale factors

Fig 2 shows the *β* dependence on age in a linear-log plot in order to better visualize the behaviour at small age values. For comparison we included data from [34]. The agreement between the two data sets is reasonable, but our data cover a wider age range than that reported so far. A novel non-monotonic behaviour is observed with an increase of *β* values in the extremes of life.

Fig 3 shows the dependence of *α*_0_, *α*_1_, and *α*_2_ (*m* = 1) on age, including data from [34].

Again the agreement between the two data sets is reasonable. In addition, the exponents exhibit a non-monotonous behaviour. The values of *α*_0_ are widely dispersed, and their calculation is discussed below;*α*_1_ and *α*_2_ have a minimum value at around one year of age.

Data in Ref [35] can be compared with *α*_0_ and *α*_1_. Though the general behaviour with age is similar, these data show more dispersion and decrease more rapidly with age, reaching values close to 0.5 for young adults. This type of deviation was observed in our data with the increase of *m*. High frequency scale factors are very sensitive to the increase of *m*.

**Fig 4:**
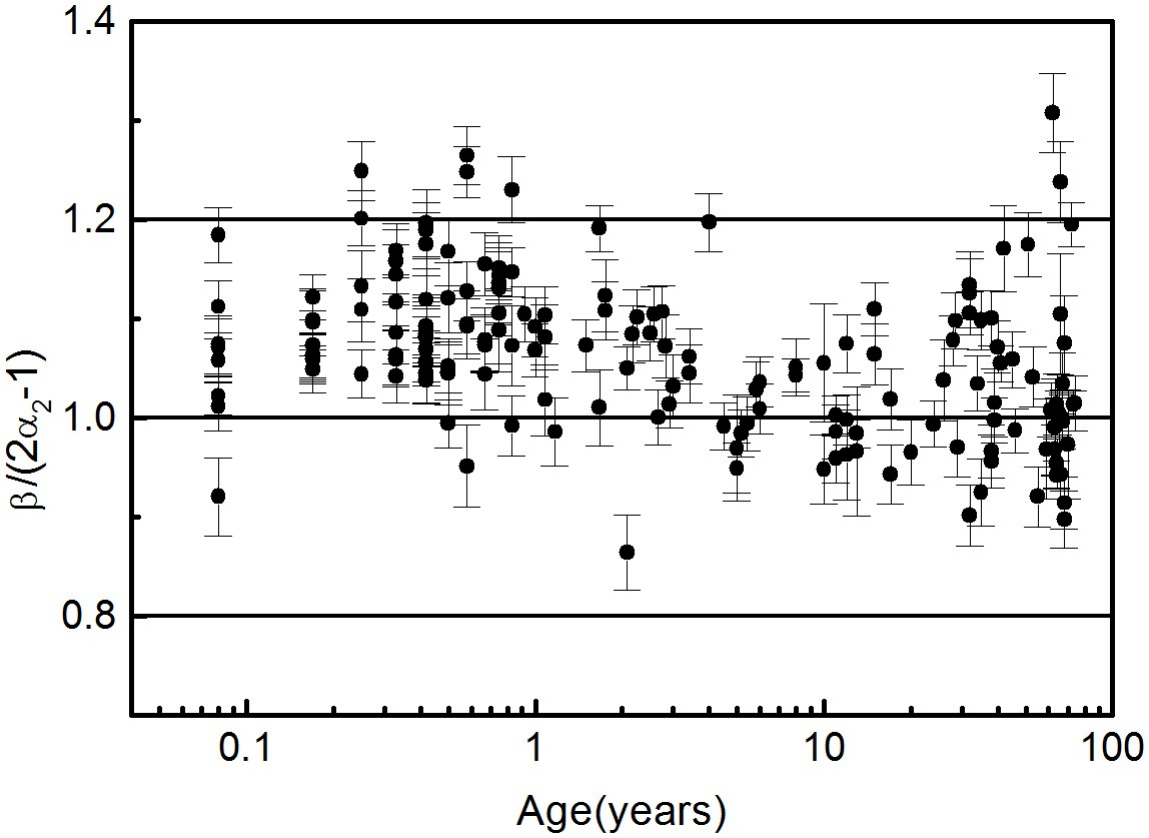
$q=\frac{\beta }{2\alpha_{2}-1}$ dependence on age.

We also analysed the relationship between *α* and *β*. Of the three scale factors, the one that correlates best with beta is *α*_2_. In Fig 4 we plot the quotient q as a function of age, being

$$\begin{array}{c} q=\frac{\beta }{2\alpha_{2}-1} \end{array}$$ (7)

If the scale relationship between *α*_2_ and *β* is fulfilled, q should be equal to 1, but the values of q deviate as age decreases. We discuss the possible causes of this discrepancy in the next section.

## Discussion and conclusions

In this work we analyse data from 24-hour Holter records for a total of 700 healthy subjects, 154 of whom were volunteers recruited for this study. The analysis covers the largest age range analysed so far. As we mentioned before, many other works in the literature consider HRV dependence on age and gender, covering an age range of 1-99 years [2, 33–44]. Our work increases the age range by slightly more than a power of ten—a significant difference for detecting scaling behaviour. As we mentioned in the Introduction, Finley et al. [43] also reported data for ages as early as 1 month. Of the indices calculated in their work, total power is comparable to HRV standard deviation (see ii). It should be noted that in [43] the records analysed have a length of 4 h during sleep and 1 h during wakefulness. According to the quality criteria established in [47, 48], those series are too short to ensure the robustness of the calculations (that is, to produce results independent of the length of the series). Only the longest period could then be used for a qualitative comparison with our work. Another important methodological difference is the report of the results grouped by age ranges, that is, the consideration of age as a discrete variable instead of a continuous one. Van den Berg et al. [44] also presented a very large study that included an age range from 1 month, but it was performed on 10-second electrocardiograms, which are too short to allow calculating *α* and *β* indices. The use of short records is undoubtedly desirable for their practical utility, but the length of the series also affects their frequency composition, and therefore the comparison must be careful, as discussed below.

We calculate indices in the time domain: <*RR*>, *SD*_*RR*_, *rMSSD*_*RR*_, and *pNN*50, measurements in the frequency domain (*β*) and multifractal scale fractals (*α*).

Our work is limited to the analysis of the above-mentioned HRV measurements; however, the methodological aspects presented in this work are useful to analyse any other measurements.

Our main conclusions are the following:

(i)<*RR*> follows a scaling relationship with age, which is independent of gender. This power-law behaviour reveals a self-similar growth process, which can be related to the basal metabolic rate (BMR) [64–70]. Allometric relationships for <*RR*> with body mass (BM) or the BMR through Kleiber’s law can be written as:
  $$\begin{array}{c} <RR>=b{\left(BM\right)}^{a} \end{array}$$ (8)
  $$\begin{array}{c} <RR>=c{\left(BMR\right)}^{d} \end{array}$$ (9)
  where it has been determined that *a* = 1/4 for mammalians, and the value of *d*depends on the scale factor in the Kleiber’s law [65]. The *d* value was initially established equal to 3/4, though it has recently been questioned based on empirical evidence that showed a wealth of robust exponents deviating from 3/4 [69, 70]. In Eq (3) the scale relationship is established with age with a scale factor of ∼1/8.

An additional important aspect is that this scaling relationship remains unchanged throughout life. One should expect deviations at an even earlier age, and it would therefore be interesting to analyse the validity of this relationship from conception and not from birth.

(ii) Statistical measures such as *SD*_*RR*_, *rMSSD*_*RR*_, and *pNN*50 show an abrupt change at the age of 12 years. For simplicity we assume the same cut-off (independent of gender) for all measures, but this assumption should be further studied. The threshold *x* = 12 was then determined for *SD*_*RR*_ looking for the best the linear fits on both sides of the threshold. Note in Fig 1 that data reported in [34] already indicated the existence of a maximum. But to attribute this maximum to a change in a scale behaviour requires analysing wide age ranges and using age as a continuous variable, as was done in this work. Quadratic fits over the complete data have been tested, but they were statistically less reliable than those presented in this work. Regarding data reported for HRV during sleep in [43], Finley et al. distinguish between quiet and active states. The maximum is clearly observed in quiet sleep between the ages of 6 and 11, while in the active state there is a continuous decrease. To compare with our work, it is appropriate to consider the two states together, and the maximum then occurs near the age of 11 years. However, a closer comparison is not possible due to the methodological differences mentioned above.

Changes of *SD*_*RR*_, *rMSSD*_*RR*_, and *pNN*50 at the age of 12 years are related to HRV changes at high frequencies, i.e. to alterations in the frequency composition of HRV. We note that the scale law of <*RR*> does not change, while those of the other measures do, i.e. over time there is a redistribution at lower frequencies, without altering the mean value of the series.

An increase in individual variation is also observed above 12 years of age, which continues even when discriminating by gender. As we mentioned in the results, this large individual variation, which is manifested in low R values, cannot be attributed to the differences between the studies used in this work because they were also observed in each of these studies. On the other hand, a large interindividual variation of autonomic function has also been described in healthy subjects [71–74]. If a process of redistribution does indeed occur in the frequency composition of HRV with age, then individual changes in the onset and progression of this process may explain the observed variability. In [6, 7] the authors studied the influence of both the ANS and the SAN receptors on HRV. They found that the deterioration of mechanisms intrinsic to the SAN leads to an increased <*RR*> and HRV. The extrinsic mechanisms (ANS input) compensate for deteriorated intrinsic mechanisms to preserve <RR>, but this compensation is associated with a reduction in the HRV.

Below the age of 12, results are gender-independent, while above that age, there seems to be a slight dependence on gender. The results suggest that the frequency composition of HRV is also altered. In fact, this alteration seems to occur at high frequencies because *β* values, which are determined at low frequencies, do not depend on gender (see below). Studies in [16] reported sex- differences in the HRV under stress. The authors suggested that men and women have different autonomic “strategies” for dealing with stress, such that men rely more on the hypothalamic-pituitary-adrenal (HPA) axis activation, and women on parasympathetic withdrawal. The researchers did not report differences in the baseline state (without stress), but these differences could not be detected due to the age range (18-60 years) and the length of the records used. We believe that we are measuring a baseline state, the one that was not detected in [16].

There are studies that support a potential protective role of estradiol in the parasympathetic cardiac tone [17, 18]. The effect of progesterone and its potent metabolite allopregnanolone on ANS is uncertain. Our study was not controlled for menstrual cycle phase. We think that the differences found in this work are beyond those that can be attributed to the cycle by the age range and the magnitude of the population analysed, and by the diversity of the origins of the information. In other words, the effects of the cycle phases should be averaged.

Finally, if the length of the electrocardiographic records limits the frequency range that can be analysed, and this range is also influenced by gender, it is clear that long and short records will differ in the information they provide and also in their clinical utility. Even though short records may not necessarily replace long ones in all applications, additional understanding of the information provided by each type would be necessary.

(iii) We performed a unified analysis of the scale factors (*α* and *β*) derived from both the multifractal and the spectral power analyses. Because of the mathematical relationship between these factors, the calculation was done by two independent methods. Whereas for the calculation of *α* we respected the scale ranges used in the literature, the method developed to calculate *β* used the optimal range for each participant. Of the three scale factors derived from the multifractal analysis, the one that correlates best with *β* is *α*_2_. Although the relationship between *α* and *β* can be then verified reasonably well, at least for people older than 5, there is a systematic deviation at an earlier age because the scale ranges are age-dependent. But more importantly, as a methodological change, the scaling hypothesis must be tested in the same range in both the time and the frequency domains. Therefore the determination of the scale factors must be based on simultaneous and independent calculations of *α* and *β*. The scale factors *α* and *β* exhibit a non-monotonic relationship with age, increasing in the extremes of life and reaching a minimum at around one year of age. This behaviour, evident in *α*_1_ and *α*_2_, has not been reported before. The values of *α*_0_ are widely dispersed due to the narrow scale range (5 < *s* < 10) used in the calculation. In this scale range, a certain dependence on gender should be evidenced, but the dispersion of the results prevents noticing it. We attribute the changes in *α*_2_ and *β* to the neuronal control on the HRV. As they are calculated at low frequencies (long-range scale), hormonal effects are negligible, and results are gender-independent. In [47, 48] we showed that an increase in *β* values can be attributed to a predominance of the sympathetic system over the parasympathetic one. At early ages, this predominance is due to the lack of maturation of the parasympathetic modulation, while at older ages it is explained by a decrease in the parasympathetic modulations.

Our knowledge of the population characteristics is limited, we do not have information on the menstrual status of all women, the socioeconomic status of the population, the BMI, etc. All studies met minimum requirements, patients were examined by clinicians and minimum quality requirements were established on Holter records and time series. However, the clinical normality criteria were left to the intervening physicians and our study was based solely on the analysis of Holter records. Our results do not allow us to distinguish the contributions of ANS and SAN [6, 7]. In this context, the sympathetic and parasympathetic modulations mentioned above are those detected at the output of the SAN.

In summary, we can establish basic scale relationships in different HRV measurements performed on long records. Above the age of 12, there is a gender dependence that mainly alters the high frequency region. Changes attributable to neuronal activity (including SAN) are observed at low frequencies. These differences must be considered when interpreting measurements based on recordings of a few seconds.

The scale factors should be calculated individually in age-dependent scale ranges and equally in both time and frequency domains.

In [75] we presented the basis for the development of implantable devices that mimic RR variability of the healthy heart. The present work has direct implications for that project that is now in progress, because that development requires a comprehensive characterization of HRV, such as the one this study presents.
